# Sociodemographic Factors and Implant Consideration by Patients Attending Removable Prosthodontics Clinics

**DOI:** 10.1155/2022/8466979

**Published:** 2022-07-11

**Authors:** Rasha A. Alamoush, Wijdan R. Elmanaseer, Yasmine W. Matar, Salah Al-Omoush, Julian D. Satterthwaite

**Affiliations:** ^1^Department of Prosthodontics, School of Dentistry, The University of Jordan, Amman 11942, Jordan; ^2^Jordan University Hospital, The University of Jordan, Amman 11942, Jordan; ^3^School of Medical Sciences, The University of Manchester, Manchester, UK

## Abstract

**Objective:**

This study is aimed at investigating the treatment options offered to and chosen by patients attending a student prosthodontics clinic and to investigate the effect of the sociodemographic background of participants on implant consideration. *Material and Methods*. A cross-sectional descriptive study was conducted on 200 participants including their socioeconomic background, treatment options presented, treatment preferences, and implant consideration. Bivariate tests (unpaired *t*-test, chi-square, and Mann–Whitney test) and Spearman correlation were used for comparison of different socioeconomic groups according to treatment preferences (implant consideration versus conventional removable prosthesis).

**Results:**

Treatment options presented by dental students include 63.5% complete denture, 32% implants/removable dentures, and partial denture 4.5%. Conventional removable prostheses were mostly chosen due to low income. Implants were only considered by 26% of participants. Age and implant consideration had a significant negative correlation. No significant difference in gender and residency area on implant consideration was found. The chi-square test showed a significant difference between implant consideration and conventional removable prostheses in the various occupation groups.

**Conclusions:**

Low income is the main factor prohibiting patients from considering dental implants. Age and educational level may play a considerable role in considering dental implants. There should be more emphasis on dental students' treatment planning education to include and explain dental implants as a treatment option for their patients in the prosthodontics clinic.

## 1. Introduction

Maintaining teeth throughout life is considered an important reflection of oral health. Nevertheless, edentulism is very common among older people, and it can affect their health and life quality [1–3]. Although tooth loss is mainly caused by dental caries and periodontal disease, sociodemographic factors and lifestyles are directly correlated to tooth loss and also to the subsequent treatment options considered [4–7]. Dental attendance, health care systems, educational levels, income, and oral hygiene are all factors that contribute to tooth loss [5, 6]. Additionally, age, gender, and rural residency are also factors that contribute to tooth loss, which can negatively influence the self-esteem, socialization, and daily life of patients [8, 9], with loss of chewing function and poor aesthetics associated with tooth loss [10, 11].

Different treatment options may be considered for edentulous and partially edentulous patients. Treatment options available for edentulous or partially edentulous patients could be removable or fixed prostheses. Removable prostheses include a removable partial denture, complete denture, and overdenture. Other treatment options include fixed prostheses such as crowns, bridges, and implants [12]. Conventional removable partial or complete dentures are associated with many problems. Elderly people experience eating, social interaction, and communication problems with their complete dentures and subsequently negative impact on their quality of life. Further, about 25% of removable prostheses are replaced or not used after 5 years of follow-up, and the percentage increased up to 50% after 10 years [13, 14].

Implant-supported or implant-retained appliances offer a more beneficial treatment than conventional ones for both edentulous and partially edentulous patients [15–17]. An implant-supported/retained prosthesis is considered a favourable treatment option, especially for patients who are less satisfied with their lower dentures. The efficacy of implant-supported lower dentures has been clearly stated in the McGill symposium [18] and is associated with better oral health status [19–22]. Therefore, dental practitioners should consider presenting implants as a predictable treatment option [23]. Nevertheless, patient-related factors play a significant role in the choice of treatment and is considerably affected by the socioeconomic background of the patients.

A study of the need and demand for complete dentures in Jordanians showed a significant relationship between sociodemographic variables and edentulism [24]. However, there has been no report on the influence of these factors on treatment choice and implant consideration. The aims of this study were (I) to investigate the treatment options presented to and chosen by the patients attending student prosthodontics clinics and (II) to investigate the effect of the sociodemographic background of participants on implant consideration.

## 2. Methods

This was a cross-sectional descriptive study that was conducted in the removable prosthodontics department of the Dentistry School, University of Jordan. The study protocol was approved by the Institutional Review Board/Deanship of Scientific Research of the University of Jordan (Reference: 10/2020/9611) and all participants were provided with a written consent form. The study group included all patients who attended the removable prosthodontics department for complete or partial denture construction and consented to participation during the period from January to August 2021. Participants were included in the study if they were partially or completely edentulous and requested teeth replacement with either removable partial or complete dentures. However, participants who needed further assessment and/or more advanced treatment were excluded.

Data were collected using a 3-section face-to-face questionnaire that focused on socioeconomic factors and dental status (edentulous and partially edentulous) in the first section, treatment options presented by dental students in the second section, and implant consideration by participants in the third section. Any questionnaires that had missing information were excluded.

A trained intern dentist filled out the questionnaire based on the information provided by the participants. The following sociodemographic information was recorded: age (stratified into 4 age groups: 20-39 years, 40-59, 60-79, and 80-100), gender, residency (rural or urban), income (categorized based on world bank organization classification for Jordan income) [25], education (stratified into no education, primary, middle or high school, diploma, bachelor, and master degree), and occupation (categorized based on the International Standard Classification of Occupations) [26]. The treatment options presented by the dental students were recorded without any input/influence from the intern dentist. Then, treatment choice, reasons for the chosen treatment, and implant treatment consideration (and reasons) were recorded by the intern dentist. No prosthodontist interference or explanations were presented to participants to find out the current participants' perspectives about implants consideration.

Data were analyzed using GraphPad Prism version 8.4.3. Descriptive statistics were used to report the frequencies (number) and percentages of participant preferences for different treatment options and implant considerations. Bivariate tests (unpaired *t*-test, chi-square, Mann–Whitney test) and Spearman correlation were used for comparison of different socioeconomic groups according to treatment preferences (implant consideration versus conventional removable prosthesis).

## 3. Results

This study includes 200 individuals with an age range of 27 to 89 years, the average sample age was 57.9 years, and the standard deviation (SD) was 11.4. The highest percentage lay within 40-59 and the lowest percentage lay within 80-100 age group. 178 (89%) patients were edentulous and 22 (11%) partially edentulous.

### 3.1. Treatment Options Presented by Dental Students, Participant Treatment Choice, and Implant Consideration

Participants were presented with various treatment options by dental students; including 63.5% complete dentures, 32% implants and removable dentures, and partial dentures 4.5%. The detailed treatment options presented are shown in Table 1.

Most participants choose conventional removable prostheses, whether complete (44%) or partial (64%), due to financial reasons. 30% considered complete dentures more comfortable and easier to construct with better function and easy adaptation with 15% thinking that complete dentures were the only treatment option. Few participants (3%) considered complete dentures more aesthetic; 1% preferred removable appliances, and 1% had failed implants. Participants choose partial removable prostheses because they want to retain their teeth (9%), it was recommended by a dentist (9%) or thinking there was no other treatment option (4.5%). Other causes include recommendations by a family member (4.5%), young age (4.5%), or nonretentive previous RPD (4.5%) (Table 2).

Regarding implant consideration as a treatment option by the participants, only 26 (13%) stated they would consider implants as a treatment option due to the following reasons: 50% of the participants would consider implants if they could afford them, 42% thought it is a better option and more retentive, and 8% thought it is more stable and permanent. On the other hand, 174 (87%) stated that they prefer conventional removable dentures and would not consider implants due to the following reasons: financial (68.4%), lack of awareness about implants (7%), and the rest have various reasons such as old age (5%), fear of surgery (2.3%), and medical conditions (2.3%) (Table 3).

### 3.2. Sociodemographic Factors and Implant Consideration

Age and implant consideration had a significant negative correlation while a significant positive correlation between age and conventional removable prostheses was found (*R*^2^ = 0.97 and *P* = 0.014) (Table 4).

Gender distribution was 46 (23%) female and 154 (77%) males. Unpaired *t*-test showed a significant difference within each gender for treatment options (*P* = 0.002) and a nonsignificant difference between males and females (*P* = 0.39) (Table 4).

The patients included were from rural (55.5%) and urban (44.5%) areas. Mann–Whitney test showed a nonsignificant difference between rural and urban areas (*P* = 0.33) both in implant and conventional removable prostheses consideration (Table 4).

Many of the patients stated that they have no income (44.5%), low income (30%), or below minimum wage (18.5%); only 4% had low to middle income; and none had upper-middle or high income. A nonsignificant negative correlation between income and implant consideration was found and a nonsignificant positive correlation between income and conventional removable prostheses (*P* = 0.56), (Table 4).

Most of the patients had at least a high school educational level (54.5%) while 12.5% had no education; 16.5% had a diplomas, bachelor, or master-level degrees. A nonsignificant positive correlation between education level and implant consideration was found while a nonsignificant negative correlation between education level and implant consideration was found (*P* = 0.07), Table 5.

Various occupations were stated by the participants but the majority had no job 58.5% and 3% were housewives. The chi-square test showed a significant difference between implant consideration and conventional removable prostheses in the various occupation groups (*P* = 0.0001), Table 5.

## 4. Discussion

This study is aimed at investigating the treatment options provided by dental students in prosthodontics clinics and participants' treatment choices and implant considerations. Further, the influence of sociodemographic background on implant consideration versus a conventional removable prosthesis was investigated.

Dental health practitioners are required to inform their patients about the various treatment options available so that the patient can thus make an informed choice when deciding the treatment options in terms of aesthetic and functional expectations [27, 28]. In this study, dental students presented various treatment options to the participants, including 63.5% complete dentures, 32% implants/removable dentures, and partial dentures 4.5%. However, these percentages reflect that implants were not mentioned by the majority of dental students. This highlights a gap in enabling patient awareness about dental implants, especially in edentulous and partially edentulous patients. Given the better quality of life and nutritional status of restorations that are implant-supported/retained compared to conventional removable dentures [23, 29] and high short and long-term successful outcomes [30–34], it is of paramount importance that implants should be included and presented as a treatment choice by dentists and dental students [23, 29].

Removable prostheses remain a viable treatment option with many advantages such as lower cost, less time, and less invasive than implant-supported/retained prostheses [28, 31, 32]. Most participants considered a removable prosthesis rather than implants due to financial constraints, in line with other studies [35–37]. Dental implants are not included in medical insurance systems in most countries, although their inclusion could avoid oral health deterioration [38–40]. A nonsignificant negative correlation between income and implant consideration was found, while a nonsignificant positive correlation was found between income and conventional removable prostheses. However, it is worth mentioning that most of the patients who attended the dental student prosthodontics clinic have no or low income, which may present a bias.

Other barriers to implant prostheses generally include fear of surgery, choosing less invasive treatment, old age, easy adaptation to complete denture, and medical conditions [31, 41, 42], and these factors are still raised even when implant treatment is provided free of charge or provided at a lower cost [43].

Age and implant consideration had a significant negative correlation while a positive correlation between age and removable consideration was found (*R*^2^ = 0.97 and *P* = 0.014), indicating that implant-retained/supported prosthesis may be considered more by younger patients even though implants can be placed successfully in the elderly provided that they have favourable clinical and socioeconomic outcomes [44, 45]. This may be explained by the aforementioned factors such as fear of surgery and easy adaptation to complete denture dentures [41, 43].

Both males and females (unpaired *t*-test showed a nonsignificant difference between males and females *P* = 0.39) showed the same trend in choosing conventional removable prosthesis rather than implant-retained/supported prosthesis. Also, a nonsignificant difference between rural and urban areas (*P* = 0.33) was found both in implant and removable denture consideration, although both groups prefer conventional removable prostheses. Again, as stated earlier, this might be mainly attributed to low income, lack of awareness, and conventional removable prosthesis being less invasive and less costly than implant-supported/retained prosthesis.

The level of education could impact the patient's treatment choice [46]. There was a nonsignificant negative correlation between the education level of the participants and implant consideration (*P* = 0.07), which means patients with higher educational levels might consider implant-retained/supported prosthesis more than those with low educational levels. Nevertheless, even those with a higher educational level but low income may not consider implants due to the cost [47]. However, variability of educational levels with some categories having fewer numbers than others might be a limiting factor to concluding a direct correlation. Further, there was a significant difference between implant consideration and removable denture in the various occupation groups (*P* = 0.0001). Nevertheless, there was not a direct correlation between implant consideration and various occupations. One limitation in this regard was that due to the study taking place in the training student clinic which provides treatment at a lower cost, it is expected that the low economic group (with no job) is the majority of participants.

In addition to the bias in participant group income level, a further potential limitation of the study relates to the students' academic and practical knowledge having a direct bearing on the treatment options presented; however, the information in the study provides a general idea about the treatment planning across a whole batch of dental students in prosthodontics clinics. Patient awareness and implant consideration might differ if decisions are made from a point of increased knowledge and awareness, although this study intended to map out the current/existing perceptions of participants without further intervention.

## 5. Conclusions

Within the limitations of this study, it was concluded that:

Low income is the main factor prohibiting patients from considering dental implants.

Age and educational level play a considerable role in considering dental implants; however, gender and residency have no influence on the treatment preferences of the participants.

There should be more emphasis on treatment planning provided by dental students to include and explain dental implants as a treatment option to their patients in the prosthodontics clinic.

## Figures and Tables

**Table 1:** The frequency (number: *n*) and percentage of the treatment options presented by dental students (CD and RPD stand for complete denture and removable partial denture, respectively).

Treatment options presented	Frequency (*n*)	Percentage (%)
CD	127	63.5%
CD/implants	42	21.0%
CD/implant-retained denture	8	4.0%
CD/implant-supported denture	1	0.5%
RPD/implants	4	2.0%
RPD/CD/implants	3	1.5%
RPD/fixed/implants	4	2.0%
Transitional RPD/implants	2	1.0%
CD/RPD	1	0.5%
RPD	5	2.5%
Transitional RPD	2	1.0%
Upper RPD/lower fixed bridge	1	0.5

**Table 2:** The frequency and percentage of the treatment choices and the reason why chosen as provided by the participants (CD, RPD stand for complete denture and removable partial denture respectively).

Treatment choice	Reason	Frequency (*n*)	Percentage (%)
CD (*n* = 178)	Low income	79	44%
	No other option	27	15%
	Easier to adapt to because the patient already had one	11	6%
	More comfortable	15	8%
	Better function	11	6%
	Easier, faster and less painful than implants	15	8%
	Better cleaning	3	2%
	Mucosal inflammation and sore gums	3	2%
	Better aesthetics	5	3%
	CD due to old age	2	1%
	CD as a non-fixed appliance	1	1%
	Recommended by family	2	1%
	Recommended by a dentist	1	1%
	Broken old denture	1	1%
	Failed implants	2	1%

RPD (*n* = 22)	Low income	14	64%
	To retain his teeth	2	9%
	Recommended by a dentist	2	9%
	No other option	1	4.5%
	Non-retentive old RPD	1	4.5%
	Recommended by family	1	4.5%
	RPD due to young age	1	4.5%

**Table 3:** The frequency and percentage of implant consideration and the reason why considered as provided by the participants.

Implant consideration	Reason	Frequency (*n*)	Percentage (%)
No (*n* = 174), 87%	Income	119	68.4%
	Not aware of	12	7%
	Old age	9	5%
	Painful	7	4%
	Cannot handle and clean it or not convinced	7	4%
	Diabetic patient	4	2.3%
	Does not have enough bone	4	2.3%
	Failed implants	2	1.2%
	Cost and afraid surgery	4	2.3%
	Friend recommendation	2	1.2%
	Adapted to CD and old age	4	2.3%

Yes (*n* = 26), 13%	If can afford it	13	50%
	Better option and more retentive	11	42%
	More stable and permanent	2	8%

**Table 4:** The frequency and percentage of implant consideration versus conventional removable prostheses in each sociodemographic factor.

Sociodemographic factor	Implant consideration	Conventional removable prostheses	Total
Age			
20-39	4 (31%)	9 (69%)	13 (6.5%)
40-59	15 (16%)	81 (84%)	96 (48%)
69-79	7 (8%)	78 (92%)	85 (42.5%)
80-100	0 (0%)	6 (100%)	6 (3%)
Total	26 (13%)	174 (87%)	200 (100%)
Gender			
F	4 (9%)	42 (91%)	46 (23%)
M	22 (14%)	132 (86%)	154 (77%)
Total	26 (13%)	174 (87%)	200 (100%)
Residency			
Rural	17 (15%)	94 (85%)	111 (55.5%)
Urban	9 (10%)	80 (90%)	89 (44.5%)
Total	26 (13%)	174 (87%)	200 (100%)
Income [25]			
None	9 (10%)	80 (90%)	89 (44.5%)
Below minimum wage	5 (14%)	32 (86%)	37 (18.5%)
Low income	12 (18%)	54 (82%)	66 (33%)
Lower-middle income	0 (0%)	8 (100%)	8 (4%)
Total	26 (13%)	174 (87%)	200 (100%)

**Table 5:** The frequency and percentage of implant consideration versus conventional removable prostheses in various education levels and occupations.

Sociodemographic factor	Implant consideration	Conventional removable prostheses	Total
Education			
No education	0 (0%)	25 (100%)	25 (12.5%)
Primary school	3 (11%)	25 (89%)	28 (14%)
Middle school	1 (20%)	4 (80%)	5 (2.5%)
High school	16 (15%)	93 (85%)	109 (54.5%)
Diploma	2 (14%)	12 (86%)	14 (7%)
Bachelor	3 (17%)	15 (83%)	18 (9%)
Master	1 (100%)	0 (0%)	1 (0.5%)
Total	26 (13%)	174 (87%)	200 (100%)
Job classification [26]			
Professionals	2 (20%)	8 (80%)	10 (5%)
Technician and associate professionals	5 (28%)	13 (72%)	18 (9%)
Clerical support work	1 (33%)	2 (67%)	3 (1.5%)
Service and sales workers	1 (13%)	7 (87%)	8 (4%)
Crafts and related trade workers	1 (8%)	11 (92%)	12 (6%)
Plant and machine operators and assemblers	3 (18%)	14 (82%)	17 (8.5%)
Elementary occupation	2 (25%)	6 (75%)	8 (4%)
Armed forces occupation	1 (100%)	0 (0%)	1 (0.5%)
House wife	0 (0%)	6 (100%)	6 (3%)
None	10 (9%)	107 (91%)	117 (58.5%)
Total	26 (13%)	174 (87%)	200 (100%)

## Data Availability

The data used to support the findings of this study are included within the article.

## References

[1] Bekiroglu N., Çiftçi A., Bayraktar K., Yavuz A., Kargul B. (2012). Oral complaints of denture-wearing elderly people living in two nursing homes in Istanbul. *Oral Health and Dental Management*.

[2] Shamdol Z., Ismail N. M., Hamzah N. T., Ismail A. R. (2008). Prevalence and associated factors of edentulism among elderly Muslims in Kota Bharu, Kelantan, *Malaysia*. *America*.

[3] Vadavadagi S. V., Srinivasa H., Goutham G. B., Hajira N., Lahari M., Reddy G. T. P. (2015). Partial edentulism and its association with socio-demographic variables among subjects attending dental teaching institutions, India. *Journal of International Oral Health*.

[4] Razak P. A., Richard K. M. J., Thankachan R. P., Hafiz K. A. A., Kumar K. N., Sameer K. M. (2014). Geriatric oral health: a review article. *Journal of International Oral Health*.

[5] Donaldson A., Everitt B., Newton J., Steele J., Sherriff M., Bower E. (2008). The effects of social class and dental attendance on oral health. *Journal of Dental Research*.

[6] Cunha-Cruz J., Hujoel P. P., Nadanovsky P. (2007). Secular trends in socio-economic disparities in edentulism: USA, 1972-2001. *Journal of Dental Research*.

[7] Esan T. A., Olusile A. O., Akeredolu P. A., Esan A. O. (2004). Socio-demographic factors and edentulism: the Nigerian experience. *BMC Oral Health*.

[8] Tiwari T., Scarbro S., Bryant L. L., Puma J. (2016). Factors associated with tooth loss in older adults in rural Colorado. *Journal of Community Health*.

[9] Medina-Solís C. E., Pérez-Núñez R., Maupomé G., Casanova-Rosado J. F. (2006). Edentulism among Mexican adults aged 35 years and older and associated factors. *American Journal of Public Health*.

[10] Batista M. J., Lawrence H. P., Rosário de Sousa M. L. (2014). Impact of tooth loss related to number and position on oral health quality of life among adults. *Health and Quality of Life Outcomes*.

[11] Gerritsen A. E., Allen P. F., Witter D. J., Bronkhorst E. M., Creugers N. H. J. (2010). Tooth loss and oral health-related quality of life: a systematic review and meta-analysis. *Health and Quality of Life Outcomes*.

[12] Carr A. B., McCracken D. (2011). *Removable Partial Prosthodontics*.

[13] Hummel S. K., Wilson M. A., Marker V. A., Nunn M. E. (2002). Quality of removable partial dentures worn by the adult U.S. population. *The Journal of Prosthetic Dentistry*.

[14] Vermeulen A. H. B. M., Keltjens H. M. A. M., van’t Hof M. A., Kayser A. F. (1996). Ten-year evaluation of removable partial dentures: survival rates based on retreatment, not wearing and replacement. *The Journal of Prosthetic Dentistry*.

[15] Misch C. E. (2004). *Dental implant prosthetics-E-book*.

[16] Zhang H., Ramos V., Bratos M., Liu P. P., He W. (2021). Effect of the attachments on clinical outcomes of mandibular distal extension implant-supported removable partial dentures: a systematic review. *The Journal of Prosthetic Dentistry*.

[17] Zancopé K., Abrão G. M., Karam F. K., Neves F. D. (2015). Placement of a distal implant to convert a mandibular removable Kennedy class I to an implant-supported partial removable class III dental prosthesis: a systematic review. *The Journal of Prosthetic Dentistry*.

[18] Feine J. S., Carlsson G. E., Awad M. A. (2002). The McGill consensus statement on overdentures. Montreal, Quebec, Canada. May 24-25, 2002. *The International Journal of Prosthodontics*.

[19] Awad M. A., Lund J. P., Shapiro S. H. (2003). Oral health status and treatment satisfaction with mandibular implant overdentures and conventional dentures: a randomized clinical trial in a senior population. *International Journal of Prosthodontics*.

[20] Thomason J. M., Lund J. P., Chehade A., Feine J. S. (2003). Patient satisfaction with mandibular implant overdentures and conventional dentures 6 months after delivery. *International Journal of Prosthodontics*.

[21] Siadat H., Alikhasi M., Mirfazaelian A., Geramipanah F., Zaery F. (2008). Patient satisfaction with implant-retained mandibular overdentures: a retrospective study. *Clinical Implant Dentistry and Related Research*.

[22] Kuoppala R., Näpänkangas R., Raustia A. (2013). Quality of life of patients treated with implant-supported mandibular overdentures evaluated with the oral health impact profile (OHIP-14): a survey of 58 patients. *Journal of Oral & Maxillofacial Research*.

[23] Turkyilmaz I., Company A., McGlumphy E. (2009). Should edentulous patients be constrained to removable complete dentures? The use of dental implants to improve quality of life for edentulous patients. *Gerodontology*.

[24] Al-Dwairi P. Z. (2010). Complete edentulism and socioeconomic factors in a Jordanian population. *The International Journal of Prosthodontics*.

[25] https://www.worldbank.org/en/country/jordan/brief/qa-jordan-country-reclassification.

[26] ISCO–08 (2012). *International Standard Classification of Occupations*.

[27] Rich B., Goldstein G. R. (2002). New paradigms in prosthodontic treatment planning: a literature review. *The Journal of Prosthetic Dentistry*.

[28] Shrira N. D., Deshmukh S. P., Pande N. A., Radke U. M. (2016). An evaluation of patient's decisions regarding dental prosthetic treatment. *The Journal of the Indian Prosthodontic Society*.

[29] El Osta N., El Osta L., Moukaddem F. (2017). Impact of implant-supported prostheses on nutritional status and oral health perception in edentulous patients. *Clinical Nutrition ESPEN*.

[30] Kim J. J. (2019). Revisiting the removable partial denture. *Dental Clinics of North America*.

[31] Lang N. P., Zitzmann N. U., on behalf of Working Group 3 of the VIII European Workshop on Periodontology (2012). Clinical research in implant dentistry: evaluation of implant-supported restorations, aesthetic and patient-reported outcomes. *Journal of Clinical Periodontology*.

[32] Papi P., Di Carlo S., Mencio F., Rosella D., De Angelis F., Pompa G. (2017). Dental implants placed in patients with mechanical risk factors: a long-term follow-up retrospective study. *Journal of International Society of Preventive and Community Dentistry*.

[33] Berglundh T., Persson L., Klinge B. (2002). A systematic review of the incidence of biological and technical complications in implant dentistry reported in prospective longitudinal studies of at least 5 years. *Journal of Clinical Periodontology*.

[34] D’Addazio G., Xhajanka E., Cerone P. (2021). Traditional removable partial dentures versus implant-supported removable partial dentures: A retrospective, observational oral health-related quality-of-life study. *Prosthesis*.

[35] Ellis J. S., Levine A., Bedos C. (2011). Refusal of implant supported mandibular overdentures by elderly patients. *Gerodontology*.

[36] Narby B., Kronström M., Söderfeldt B., Palmqvist S. (2008). Changes in attitudes toward desire for implant treatment: a longitudinal study of a middle-aged and older Swedish population. *International Journal of Prosthodontics*.

[37] AL-Dwairi Z. N., El Masoud B. M., AL-Afifi S. A., Borzabadi-Farahani A., Lynch E. (2014). Awareness, attitude, and expectations toward dental implants among removable prostheses wearers. *Journal of Prosthodontics*.

[38] Choi J.-S., Ma D.-S. (2020). The financial estimate of dental implant treatment about the National Health Insurance coverage for the Korean young adults. *Journal of Korean Academy of Oral Health*.

[39] Carlsson G. E., Kronström M., de Baat C. (2004). A survey of the use of mandibular implant overdentures in 10 countries. *The Journal of Prosthetic Dentistry*.

[40] Lee K., Dam C., Huh J., Park K.-M., Kim S.-Y., Park W. (2017). Distribution of medical status and medications in elderly patients treated with dental implant surgery covered by national healthcare insurance in Korea. *Journal of Dental Anesthesia and Pain Medicine.*.

[41] Leles C. R., Ferreira N. P., Vieira A. H., Campos A. C., Silva E. T. (2011). Factors influencing edentulous patients’ preferences for prosthodontic treatment. *Journal of Oral Rehabilitation*.

[42] Papi P., Giardino R., Sassano P., Amodeo G., Pompa G., Cascone P. (2015). Oral health related quality of life in cleft lip and palate patients rehabilitated with conventional prostheses or dental implants. *Journal of International Society of Preventive & Community Dentistry*.

[43] Walton J. N., MacEntee M. I. (2005). Choosing or refusing oral implants: a prospective study of edentulous volunteers for a clinical trial. *The Journal of Prosthetic Dentistry*.

[44] Bartold P. M., Ivanovski S., Darby I. (2016). Implants for the aged patient: biological, clinical and sociological considerations. *Periodontology 2000*.

[45] Becker W., Hujoel P., Becker B. E., Wohrle P. (2016). Dental implants in an aged population: evaluation of periodontal health, bone loss, implant survival, and quality of life. *Clinical Implant Dentistry and Related Research.*.

[46] Leles C. R., Dias D. R., Nogueira T. E., McKenna G., Schimmel M., Jordão L. M. R. (2019). Impact of patient characteristics on edentulous subjects’ preferences for prosthodontic rehabilitation with implants. *Clinical Oral Implants Research*.

[47] Abbas H., Aida J., Saito M. (2019). Income or education, which has a stronger association with dental implant use in elderly people in Japan?. *International Dental Journal*.

